# Association of an HIV-Prediction Model with Uptake of Preexposure Prophylaxis

**DOI:** 10.1055/a-2524-4993

**Published:** 2025-06-04

**Authors:** Steven Romero, Kristin S. Alvarez, Ank E. Nijhawan, Arun Nethi, Katie Bistransin, Helen L. King

**Affiliations:** 1 Department of Pharmacy, UT Southwestern Medical Center, Dallas TX and Parkland Health, Dallas, Texas, United States; 2 Center of Innovation and Value at Parkland, Parkland Health, Dallas, Texas, United States; 3 Division of Infectious Diseases and Geographic Medicine, Department of Internal Medicine, UT Southwestern Medical Center, Dallas TX and Parkland Health, Dallas, Texas, United States; 4 Parkland Center for Clinical Innovation, Dallas, Texas, United States; 5 Department of Pharmacy, Parkland Health, Dallas, Texas, United States

**Keywords:** machine learning, human immunodeficiency virus, preexposure prophylaxis, decision support systems

## Abstract

**Background:**

Global efforts aimed at ending human immunodeficiency virus (HIV) incidence have adapted and evolved since the turn of the century. The utilization of machine learning incorporated into an electronic health record (EHR) can be refined into prediction models that identify when an individual is at greater HIV infection risk. This can create a novel and innovative approach to identifying patients eligible for preventative therapy.

**Objectives:**

This study's aim was to evaluate the effectiveness of an HIV prediction model in clinical workflows. Outcomes included preexposure prophylaxis (PrEP) prescriptions generated and the model's ability to identify eligible patients.

**Methods:**

A prediction model was developed and implemented at the safety-net hospital in Dallas County. Patients seen in primary care clinics were evaluated between July 2020 and June 2022. The prediction model was incorporated into an existing best practice advisory (BPA) used to identify potentially eligible PrEP patients. The prior, basic BPA (bBPA) displayed if a prior sexually transmitted infection was documented, and the enhanced BPA (eBPA) incorporated the HIV prediction model.

**Results:**

A total of 3,218 unique patients received the BPA during the study time period, with 2,346 ultimately included for evaluation. There were 678 patients in the bBPA group and 1,666 in the eBPA group. PrEP prescriptions generated increased in the postimplementation group within the 90-day follow-up period (bBPA:1.48 vs. eBPA:3.67 prescriptions per month, *p* < 0.001). Patient demographics also differed between groups, resulting in a higher median age (bBPA: 36[interquartile range (IQR): 24] vs. eBPA: 52[QR: 19] years, *p* < 0.001) and an even distribution between birth sex in the postimplementation group (female sex at birth bBPA: 62.2% vs. eBPA:50.2%, *p* ≤ 0.001).

**Conclusion:**

The implementation of an HIV prediction model yielded a higher number of PrEP prescriptions generated and was associated with the identification of twice the number of potentially eligible patients.

## Background and Significance

Despite great advancements in the prevention and treatment of human immunodeficiency virus (HIV), the incidence of infection persists at high levels. In 2021, there were 36,000 individuals diagnosed with HIV in the United States. 1 The national Ending the HIV Epidemic (EHE) initiative aims to reduce new HIV diagnoses in the United States by 90% by 2030, and one strategy to reach this goal is to increase the uptake of HIV preexposure prophylaxis (PrEP) among individuals at increased likelihood of acquiring HIV. 2 The EHE goal is for 50% of eligible patients to be prescribed and maintained on PrEP. 3 Currently, the CDC estimates that only 30% of individuals eligible for PrEP in the United States have been prescribed PrEP, resulting in a substantial mismatch between need and PrEP coverage by region. 1 3 Though the highest HIV incidence is seen in the southern region of the United States, accounting for 52% of new diagnoses in 2021, the South only accounted for 38% of PrEP users in 2022. 4 Dallas County, specifically, is a priority jurisdiction of the EHE efforts. This highlights that despite some improvements in PrEP uptake nationally, these interventions are not reaching populations that would most benefit, further exacerbating health disparities. Strategies to identify patients who would benefit from PrEP and improve prescribing are essential in the efforts to end the HIV epidemic.

The U.S. Preventative Services Task Force (USPSTF) recommends clinicians offer PrEP to individuals who are considered at increased risk of HIV acquisition (grade A recommendation), and the CDC maintains PrEP clinical practice guidelines to guide providers in identifying eligible patients and prescribing PrEP. 5 6 There are currently three FDA-approved regimens for PrEP that are up to 99% effective at preventing HIV, including two daily tablet regimens, emtricitabine/tenofovir disoproxil fumarate, and emtricitabine/tenofovir alafenamide, and the long-acting intramuscular cabotegravir. Despite these resources, PrEP has been vastly under-utilized, especially by primary care providers (PCPs). Barriers identified by PCPs to providing PrEP include limited knowledge of PrEP prescribing, time to assess eligibility and counsel on risk reduction, and concerns over PrEP costs. 7 8 9 These barriers must be addressed to improve the uptake of PrEP prescribing among PCPs. 9

Advancements in clinical decision support systems (CDSS) technology provide an opportunity to overcome some of these barriers. Such technological interventions have displayed efficiency in equipping providers with tools to better utilize disease-preventing therapies as well as suggesting an improvement in patient quality of life. 10 11 The implementation of machine learning to develop prediction models that aid in identifying individuals at increased risk of HIV infection has come to the forefront of prevention medicine. Health systems have developed prediction models using health record data to help identify such individuals in their respective patient populations. 12 13 14 Additional models have been described, expanding to wider populations, including cisgender women and the Southern United States. 15 16 However, there has been limited assessment of the prospective implementation of such models into clinical practice and the evaluation of their impact on PrEP prescribing. 17 18

Parkland Health in Dallas, Texas, a county-funded health system, has internally developed and validated an HIV prediction model. 14 In this study, we aimed to analyze the association between incorporating this HIV prediction model into an existing best practice advisory (BPA) and PrEP prescribing. Specifically, we compared: (1) the frequency of BPA alerts; (2) the population identified by the BPA; and (3) the number of PrEP prescriptions before and after prediction model implementation.

## Methods

### Study Setting, Population, and Design

Our study setting, Parkland Health in Dallas, Texas, is a comprehensive, county-funded healthcare system that serves approximately one million patient visits annually. 19 Parkland Health uses an electronic health record (EHR), EPIC (Verona, WI), to document both inpatient and ambulatory healthcare data. A prediction model was internally developed and validated to predict the risk of incident HIV infection for any patient 16 years or older between 2015 and 2019. 14

### Intervention

**Fig. 1:**
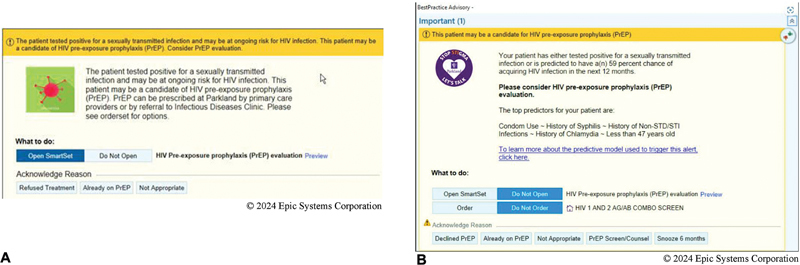
( **A** ) Original basic PrEP BPA (bBPA) displaying only when a positive bacterial STI was documented in the preceding 180 days. ( **B** ) Enhanced PrEP BPA (eBPA) including a display if the HIV risk score was ≥70%.

A basic PrEP BPA (bBPA) that alerts PCPs if their patient may benefit from HIV PrEP during their clinic visit was incorporated into the EHR in July 2020. The bBPA appeared upon chart opening when a patient had tested positive for a bacterial sexually transmitted infection (STI) in the previous 6 months, including positive chlamydia (CT), gonorrhea (GC), or syphilis test ( Fig. 1A ). Options for resolution of the bBPA were: refused treatment, already on PrEP, Not Appropriate. An enhancement to this PrEP BPA (eBPA) additionally incorporated a validated HIV prediction score; computed via the internally developed prediction model. 14 The eBPA would appear based on the same criteria as the bBPA or if a patient was predicted to be at increased likelihood of HIV in the subsequent year as determined by the model. Also, the PCP was given two additional options to resolve the BPA ( Fig. 1B ). In both the bBPA and eBPA a link was provided to a provider prescribing guide, patient information sheets, prepopulated laboratory and medication orders, and clinic note templates. Implementation of this intervention was paired with provider education, including a well-attended provider education series on sexual health and information provided at all staff meetings. 20

### HIV Prediction Model

The Parkland Center for Clinical Innovation's (PCCI) HIV prediction model is a machine learning model, built using a light gradient boosting machine (LGBM) algorithm. LGBM is an ensemble of decision trees trained sequentially one after the other, improving from the errors of the predecessor to result in a strong boosting classifier. 14 Overall, this model uses 26 input variables to predict the individuals at increased likelihood of acquiring HIV. The prediction model, when evaluated using an unseen validation dataset, was previously found to classify the patient population of HIV and non-HIV with an AUC of 0.85. 14

### Inclusion and Exclusion Criteria

All Parkland Health patients 16 years or older, seen in primary care clinics, between July 2020 through June 2022 were included in this study. The bBPA was active from July 1, 2020, to March 31, 2022, whereas the eBPA with HIV prediction scoring was active between April 1 and June 30, 2022. One BPA occurrence was needed for a patient to meet inclusion criteria and recurring BPAs on the same patient were not considered as an additional index BPA.

Patients were excluded if any of the following criteria were met: a BPA occurred in both the pre- and postenhancement time frames, a confirmed HIV diagnosis was documented in the EHR on or prior to the date of the first BPA, calculated creatinine clearance (CrCl) was less than 60 mL/min for female and less than 30 mL/min for male (due to institutional formulary options for PrEP at the time of this study), if data was not available to calculate CrCl, or if the patient was deceased within 3 months of first BPA.

Variables collected for individual patients included: patient demographic data, sexual orientation, preferred language, and payor status. Also, STI screening information was obtained initially as a form of demographic data and a measured outcome of the intervention.

### Outcomes

Our primary outcome was the number of PrEP prescriptions (RXs) over 90 days after the BPA was displayed, comparing pre- and post-BPA model enhancement. Secondary outcomes included remaining comprehensive prevention services (CPS) activities, including documented patient counseling on PrEP and/or patient counseling on condom use. In addition, RX-specific outcomes included PrEP adherence at 180 days after the first prescription fill. Additionally, to address the possible effect of the COVID-19 pandemic on outcomes, a sensitivity analysis was performed assessing BPA firing and PrEP RXs only in the 3 months prior to the eBPA compared with the 3 months post-eBPA intervention.

BPA occurrences were also captured to analyze the performance of the BPA in the EHR. Finally, STI laboratory orders and positivity rates were also captured to assess changes in screening efforts for other STIs. Mean values for STI laboratory orders and positivity rates per month BPA was active were compared between BPA types, and efficiency was compared by calculating the proportion of individuals with a given BPA per month who had STI laboratories ordered or tested positive.

### Statistical Analysis

The primary endpoint of RXs was adjusted for months of exposure for each BPA. The endpoints were calculated as average per month for the total exposure period. For the bBPA, there was a total of 21 months of exposure and for the eBPA, there were 3 months of exposure. Counseling for CPS, STI orders, and STI positivity were calculated in the same manner. To calculate medication adherence the proportion of days covered (PDC) method was used. All categorical and continuous variables were compared with the chi-squared test and *t* -test, respectively.

## Results

**Fig. 2:**
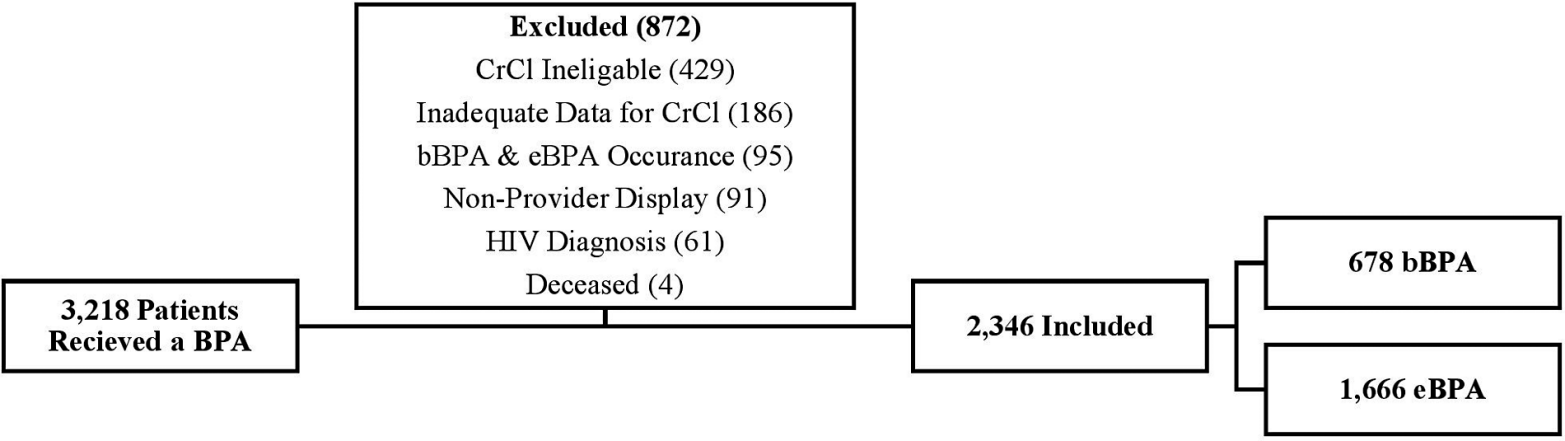
Patient flow diagram for the basic and enhanced BPA cohort inclusion.

Of the 3,218 unique BPAs that occurred between July 1, 2020, and June 30, 2022, 872 were excluded from the final analysis. Six-hundred and fifteen (19%) were ineligible due to CrCl (i.e., <60 mL/min for females and <30 mL/min for males) or did not have adequate data to calculate a CrCl, 95 (3.0%) patients had both a bBPA and eBPA fire, 91 (2.8%) displayed to non-provider staff, 61 (1.9%) had an HIV diagnosis, and 4 (0.1%) were deceased at 90-days post BPA. The final cohort included 2,346 unique patients with 678 patients in the bBPA cohort and 1,666 patients in the eBPA cohort ( Fig. 2 ).

### Demographic Data

There were several notable differences between those identified as potentially PrEP eligible by each of the BPAs ( Table 1 ). The median age for the bBPA cohort was significantly lower compared with the eBPA (36 [interquartile range (IQR): 25] vs. 52 [IQR: 19] years, respectively, *p* < 0.001) and there was significantly more female (sex at birth) patients in the bBPA group than the eBPA group (421/678 [62.2%] vs. 836/1,666 [50.2%], *p* < 0.001). Most patients did not have documentation of sexual orientation in either group or to a greater extent in the eBPA group (bBPA: 497/678 [73.3%] vs. eBPA: 1,379/1,666 [82.7%], *p* < 0.001).

**Table 1:** Baseline demographics

	Basic PrEP BPA *n* = 678	Enhanced PrEP BPA *n* = 1,666	*p*
Age at time of BPA (median, IQR; mean [SD])	36, 25 (36 [15.03])	52, 19 (50 [13.28])	0.00
Gender— *n* (%)			2.0 × 10 ^−7^
Female	421 (62.2)	836 (50.2)	
Male	257 (37.8)	830 (49.8)	
Gender identity— *n* (%)			9.9 × 10 ^−6^
Female	367 (54.1)	717 (43.0)	
Male	210 (30.9)	679 (40.9)	
Transgender female	3 (0.5)	2 (0.1)	
Transgender male	1 (0.1)	4 (0.2)	
Other	1 (0.1)	0 (0)	
Not asked	96 (14.3)	264 (15.8)	
Sexual orientation— *n* (%)			3.9 × 10 ^−6^
Bisexual	11 (1.6)	14 (0.9)	
Not disclosed	2 (0.3)	5 (0.3)	
Gay	6 (0.9)	4 (0.3)	
Lesbian	2 (0.3)	0 (0)	
Something else	0 (0)	2 (0.1)	
Straight (not lesbian or gay)	160 (23.6)	262 (15.7)	
Not asked/not documented	497 (73.3)	1,379 (82.7)	
Ethnicity-race— *n* (%)			3.4 × 10 ^−6^
Hispanic			
White	270 (39.8)	522 (31.3)	
Other	3 (0.4)	3 (0.4)	
Non-Hispanic			
Black	319 (47.1)	789 (47.3)	
White	71 (10.5)	300 (18.0)	
Asian	4 (0.6)	28 (1.6)	
Other	11 (1.6)	24 (1.4)	
Preferred language— *n* (%)			6.4 × 10 ^−6^
English	567 (83.7)	1,252 (75.2)	
Spanish	110 (16.2)	389 (23.3)	
Other	1 (0.1)	25 (1.5)	
Payor status (at time of encounter)— *n* (%)			1.7 × 10 ^−24^
Charity	390 (57.6)	1,023 (61.4)	
Medicaid	149 (21.9)	267 (16.0)	
Self-pay	36 (5.3)	10 (0.6)	
Medicare	26 (3.8)	235 (14.1)	
Commercial	77 (11.4)	131 (7.9)	
STI positivity (180 days prior to BPA)— *n* (%)	653 (96.3)	157 (9.4)	0.0
CT	333 (49.1)	67 (4.0)	6.7 × 10 ^−152^ 5.3 × 10 ^−44^ 4.9 × 10 ^−97^
GC	108 (15.9)	22 (1.3)	
Syphilis	258 (38.0)	77 (4.6)	

Abbreviations: BPA, best practice advisory; CT, chlamydia; GC, gonorrhea; IQR, interquartile range; PrEP, preexposure prophylaxis.

While there were similar proportions of non-Hispanic Black patients identified as potentially eligible in both groups (bBPA: 319/678 [47.1%] vs. eBPA: 789/1,666 [47.3%], *p* = 0.93), there were fewer Hispanic-White patients identified in the enhanced group (bBPA: 270/678 [39.7%] vs. eBPA: 522/1,666 [31.3%], *p* < 0.001). However, the eBPA group yielded a higher proportion of patients who preferred Spanish as their primary language ( Table 1 ).

Finally, those in the bBPA group had significantly higher rates of positive STI findings in the 180 days preceding the BPA occurrence (653/678 [96.3%] vs. 157/1,666 [9.4%], respectively, *p* < 0.001).

### PrEP Outcomes

A significantly higher average number of RXs were written per month with a total of 11 RXs (3.67/month) written within 90 days of the eBPA compared with 31 RXs (1.48/month) in the bBPA group ( *p* < 0.05). Additional sensitivity analysis assessing only 3 months prior to the eBPA, found only 2 RXs in the 3-months pre-eBPA with 98 patients receiving the bBPA compared with 11 RXs and 1,666 patients receiving the eBPA. A total of 44 patients (14.7/month) were counseled in the eBPA group and 116 patients (5.5/month) were counseled on PrEP with the bBPA ( *p* < 0.001). Similar increases in counseling rates on condom use with 85 patients (28.3/month) in the eBPA group and 194 patients (9.2/month) counseled in the bBPA group ( Table 2 ). There were no significant differences in demographic characteristics between groups of those who received RXs between both BPA exposure groups ( Table 3 ).

**Table 2:** Primary and secondary CPS outcomes in the 90-days post-BPA exposure

	Basic PrEP BPA *n* = 678 21 Exposure months	Enhanced PrEP BPA *n* = 1,666 3 Exposure months	*p*
Primary CPS outcome			
New PrEP RX (avg/exposure month)	1.48	3.67	1.4 × 10 ^−5^
Secondary CPS outcomes			
PrEP counseling (avg/exposure month)	5.5	14.7	1.6 × 10 ^−8^
Condom counseling (avg/exposure month)	9.2	28.3	2.7 × 10 ^−5^
PrEP prescription outcomes			
PrEP outcome— *n* (%)			
Not offered	463 (68.2)	1,537 (92.3)	1.3 × 10 ^−49^
RX	31 (4.6)	11 (0.7)	8.7 × 10 ^−10^
Declined	96 (14.2)	32 (1.9)	9.6 × 10 ^−32^
Previous RX or after 90 days	18 (2.7)	15 (0.9)	2.0 × 10 ^−3^
Not eligible	71 (10.5)	67 (4.0)	3.2 × 10 ^−9^

Abbreviations: BPA, best practice advisory; CPS, comprehensive prevention services; PrEP, preexposure prophylaxis.

**Table 3:** Description of patients who received PrEP prescriptions and medication-specific outcomes

	Basic PrEP BPA *n* = 678 21 Exposure months *n* = 31	Enhanced PrEP BPA *n* = 1,666 3 Exposure months *n* = 11	*p*
Age at the time of BPA (mean ± SD)	36.1 ± 13.5	37 ± 12.8	0.84
Gender— *n* (%)			0.97
Female	19 (61.3)	6 (54.5)	
Male	12 (38.7)	5 (45.4)	
Ethnicity-race— *n* (%)			
Hispanic			
White	10 (32.2)	5 (45.4)	0.67
Black	1 (3.2)	0 (0)	0.58
Non-Hispanic			
Black	13 (41.9)	5 (45.4)	0.88
White	5 (16.1)	1 (9)	0.94
Asian	0 (0)	0 (0)	–
Unknown	2 (6.5)	0 (0)	0.96
First fill (30 days of order; %)	18 (58.1)	7 (63.6)	5.2 × 10 ^−6^
6-month PDC (from date of first fill; mean ± SD)	0.29 (±0.2)	0.26 (±0.3)	0.71
Pharmacy— *n* (%)			0.75
External	6 (19.4)	1 (9)	
Internal	25 (80.6)	10 (90.1)	
Patient assistance program (%)	15 (48.4)	5 (45.4)	0.85

Abbreviations: BPA, best practice advisory; PrEP, preexposure prophylaxis.

### BPA Analysis

Although the BPA was displayed for more patients per month in the eBPA group (bBPA: 32.3/month vs. eBPA: 555.3/month), the number of times the BPA occurred per patient decreased significantly (15.7 [14.6] vs. 5.9 [6.75], *p* < 0.001). The rate of prescriptions per BPA was bBPA: 31/10,613 (0.002 RXs/BPA) and eBPA 11/9830 (0.001 RXs/BPA). In addition, risk scores were available for 624 patients in the bBPA (even though not displayed or set as criteria) and 1,391 in the eBPA group with the average HIV prediction score being higher in the eBPA group (bBPA: 46.8% vs. eBPA: 61.9%, *p* < 0.001).

### Sexually Transmitted Infections

The mean number of STI screenings ordered using the BPA exposure month and followed up to 6 months were higher in the eBPA group when adjusted for exposure time with a notable increase in HIV screening between eBPA compared with bBPA periods (CT: eBPA 72.7/month vs. bBPA 12.7/month, GC: eBPA 72.3 vs. bBPA 12.6, Syphilis: eBPA 67.3 vs. bBPA 8.5, HIV: eBPA 182.0 vs. bBPA 9.9, respectively, *p* < 0.001). However, when adjusted for the number of individuals for whom the BPA fired, the bBPA was more efficient (for both CT and GC, a mean of 13% of eBPA vs. 39% of the bBPA group underwent testing; for syphilis, it was 12% and 26%, respectively). The mean number of positive results for GC and syphilis tests per month was significantly higher in the eBPA group compared with the bBPA group (GC: 2.3 vs. 0.47, *p* < 0.001; syphilis: 11.7 vs. 2.67, respectively, *p* < 0.001) and non-significantly higher for CT eBPA compared with the bBPA group (CT: 3.0 vs. 1.57, respectively, *p* = 0.06), When adjusted for the number of patients who had the BPA fire, the STI positivity was higher among the bBPA group than the eBPA group (GC: eBPA 0.4% vs. bBPA 1.4%; syphilis eBPA 2.1% vs. bBPA 8.3%; CT: eBPA 0.5% vs. bBPA 3.1%). Notably, two patients were diagnosed with HIV after 18 months of follow-up in the bBPA group and none in the eBPA group (2 vs. 0, *p* < 0.001).

## Discussion

Implementing an HIV predictive model into an existing BPA for enhanced identification of potentially PrEP-eligible patients was associated with a significant increase in monthly PrEP prescribing. New PrEP prescriptions increased 2.5-fold per month after the eBPA was deployed. Collateral effects such as counseling patients on PrEP and condom use also increased after incorporating the HIV predictive model. Wider eligibility occurred when leveraging factors beyond STI positivity, which allowed for broadening to an older, more gender-balanced population. However, the demographics of people who were prescribed PrEP were not substantially different after eBPA implementation.

Prediction models with CDSS in the EHR can be a useful tool to prompt PCPs to consider sexual health risks, screen for HIV and other STIs, and counsel on preventative measures such as PrEP. 18 21 However, most of these models have been developed in populations that are predominantly White, insured, and live on the East and West coasts of the United States. 14 15 These areas have a more favorable PrEP-to-need ratio than in the Southern United States. 4 In this study, the HIV predictive model was implemented in a large safety-net system in Dallas, Texas; an area identified as a priority jurisdiction by the EHE that serves a diverse population comprised of uninsured and underinsured individuals. 2

As of 2020, there were 688 new HIV diagnoses in Dallas County, the overwhelming majority of which were seen in males (80.4%). 4 The original criteria of STI positivity alone resulted in a higher proportion of female patients identified by the bBPA. Our study found that the addition of the predictive model to the bBPA criteria shifted the proportion to a more balanced male and female population, while still successfully predicting HIV risk in the under-prescribed PrEP female population. The unique strength of this combined approach (eBPA = bBPA plus prediction model) is that the eBPA flags individuals who may come from demographic groups associated with recognized HIV epidemiology (e.g., males) as well as those who have been more challenging to identify as at risk for HIV by prediction models alone (e.g., females).

Likewise, 57% of patients newly diagnosed with HIV in Dallas County from 2020 were between the ages of 25 and 44, with only 16.9% between the age of 45 and 59. 4 We observed an increase in average age by which our model was able to identify patients, from 36 to 50 years of age between bBPA and eBPA groups, lending evidence that the model was able to identify individuals with risk for HIV that we may not have previously identified based on classic local epidemiology. However, among those that were prescribed PrEP, demographic data was not significantly different, perhaps highlighting additional barriers to PrEP offering or prescribing by providers or PrEP uptake by certain populations known to underutilize PrEP. 22 23

At the time of this study, a limited amount of literature existed analyzing the implementation of an HIV prediction model into clinical practice. 18 21 The majority of the current literature highlights the extent to which a model can predict incident HIV infections with respect to certain EHR criteria. An even smaller body of evidence shows a utility in emphasizing STI data to aid in identifying underrepresented populations for PrEP therapy. 24 However, a recent study spearheads exploration into the utility of implementing CDSS targeting HIV risk prediction; demonstrating an increase in PrEP initiation among providers that provide care for patients with an anticipated elevated risk of incident HIV within the next 3 years in Northern California. 1 Of note, however, Volk and colleagues observed a significant difference in interaction among providers; HIV-focused providers were more likely to have PrEP initiated compared with non-HIV-focused providers (hazard ratio [HR] = 2.59 [confidence interval (CI): 1.30–5.16] vs. 0.89 [CI: 0.59–1.35], “ *p* -interaction” <0.001). 18 Our study highlights a nearly 2.5-fold increase in PrEP initiation after prediction model implementation regardless of provider focus area. Moreover, our study setting was utilized and directly integrated into the building of the prediction model highlighting an underrepresented patient population; unique to the Southern United States and unlike previous literature.

Our finding of an increased rate of PrEP prescriptions after the implementation of the eBPA with integrated HIV prediction modeling stresses the need to find new and innovative approaches for providers to become aware of patients who are at increased risk of acquiring HIV infection. Future research opportunities would likely include longitudinal follow-up (i.e., PrEP adherence rates after 3 months of initiation), provider and patient perspectives on using the predictive model to assess risk, costs of care outcomes, model performance improvements leveraging broader input variable set like geographical risk indicators, clinical notes, and provider interaction with the intervention.

Our study has several limitations. First, the pre- and postimplementation study design makes it difficult to assess temporal trends. The use of a historical control group that had a sparsity of prescriptions written over a 21-month timeframe made an interrupted time series design infeasible. We were unable to determine if a provider truly acknowledged the BPA triggering a response to counsel the patient on PrEP unless there was clear documentation in the EHR. While more patients were prescribed PrEP after the incorporation of the predictive model into the BPA, this was at the expense of increased provider alerts with an almost eightfold increase in overall BPAs. The impact of provider alert fatigue on patient care is unclear, though there could be negative impacts on patient care and provider wellbeing. 25 26 Using machine learning models, such as artificial neural networks, may help mitigate alert fatigue in the future allowing for the possibility to adapt alerts based on predicted provider responses to the alert. 27 Furthermore, though incorporated whenever available, data on sexual orientation was missing for the majority of patients, limiting our ability to draw conclusions regarding the relationship between this variable and outcomes. Lastly, the timing of our study overlapped with the COVID-19 pandemic, which could confound our results, though a sensitivity analysis conducted with data only from 2022 showed similar results.

## Conclusion

The implementation of an HIV prediction model was effective in identifying potentially eligible PrEP patients and this was associated with linking patients to PrEP therapy within our institution. Adaptation and continued development of this prediction model along with assessment of other novel approaches to utilization in clinical practice are ongoing areas of investigation. Further studies are needed to assess the implementation of validated prediction models into clinical practice aimed at increasing PrEP uptake therapy while also attempting to mitigate alert fatigue.

## Clinical Relevance Statement

The ability to consolidate, analyze, and interpret pertinent patient information efficiently for providers to make interventions remains at the forefront of clinical innovation. This study highlights that machine learning when implemented utilizing EHR alerts, can provide innovative and novel approaches to connecting patients to comprehensive preventative services.

## Multiple-Choice Questions

1. What was one of the primary consequences of utilizing a BPA as the primary mode by which the prediction model executed communication to providers?
  - Patient discrimination discrepancies
  - Alert fatigue
  - Incorrect prescribing of medications
  - Prolonged visit times
  **Correct Answer** : The correct answer is option b. One of the primary limitations of utilizing a BPA alert to communicate the prediction model output was an overabundance of BPA alerts. As observed in this study, there were nearly eight times as many BPAs that fired between groups.
2. What was the unique benefit of using the prediction model when considering patient demographics?
  - The model was able to identify more cisgender males.
  - The model was able to reduce the number of falsely identified patients.
  - The model was able to correctly predict patients that would be adherent to PrEP therapy.
  - The model was able to identify a more evenly distributed patient population amongst birth sex.
  **Correct Answer** : The correct answer is option d. The postimplementation patient demographics observed were more evenly distributed amongst birth sex. Male and female sex at birth, 49.8 and 50.2%, respectively. This differs from the previously observed proportion of nearly two-thirds being female.
